# Preoperative CT-Guided Coil Localization of Lung Nodule Resection: A Single-Center Experience

**DOI:** 10.7759/cureus.42503

**Published:** 2023-07-26

**Authors:** Sawsan A Kadhem, Prapul Rajendran

**Affiliations:** 1 Radiology, Salmaniya Medical Complex, Manama, BHR; 2 Radiology, National University Hospital, Singapore, SGP

**Keywords:** retrospective study, safety, video-assisted thoracoscopic surgery, coil localization, computed tomography, lung nodule

## Abstract

Background: The increased use of computed tomography (CT) for lung cancer screening has led to an increase in the incidence of lung nodules. However, accurately localizing small or deep-seated nodules remains challenging and often requires image-guided techniques. CT-guided coil localization has emerged as a popular method for precise localization.

Methods: A retrospective analysis was conducted on a cohort of patients who underwent CT-guided coil localization followed by surgical resection of lung nodules at the National University Hospital, Singapore, between 2015 and 2021. The study examined the rates of successful localization, procedural complications, and postoperative length of stay. Descriptive statistics were employed for data analysis.

Results: This retrospective study included 55 patients with pulmonary nodules, of whom 76.4% had a history of previous malignancy. A total of 58 nodules were successfully localized using coil placement and distributed across various lung lobes. The average dimensions of the nodules were 8.4 ± 3.2 mm × 10.1 ± 4.0 mm, with an average distance of 10.9 ± 9.0 mm from the pleura. Surgical resection of the coiled nodules resulted in an average resected volume of 91.1 ± 76.0 cm^3^ and a resection margin of 9.5 ± 7.3 mm. Failed localization occurred in only 3.6% of cases. Procedural complications were observed in 21.8% of patients, including hemothorax, persistent air leaks, pneumothoraces, pleural effusion, pneumonia, and atelectasis, all of which were appropriately managed. The average length of postoperative hospital stay was 6.7 ± 15.9 days.

Conclusion: Preoperative CT-guided coil localization is a reliable and safe technique for accurately localizing lung nodules before surgical resection. The high success rate of this approach enables precise intraoperative planning. Despite procedural complications, their manageable nature and the absence of major adverse events highlight the safety of this technique. Incorporating CT-guided coil localization into routine clinical practice can significantly enhance surgical planning, improve patient outcomes, and optimize resource utilization.

## Introduction

Lung nodules are frequently encountered in clinical practice [1,2], with an increasing incidence due to the widespread use of computed tomography (CT) for lung cancer screening and diagnostic purposes. The accurate localization and subsequent resection of these nodules are essential for achieving optimal patient outcomes [3]. However, the precise localization of small or deep-seated pulmonary nodules can be challenging, often necessitating the use of image-guided techniques to facilitate successful surgical interventions [3].

One such technique is CT-guided coil localization, which has gained popularity in recent years [4,5]. This approach involves the percutaneous insertion of radiopaque coils into the target nodule under CT guidance, enabling real-time visualization of the coil position relative to the nodule and surrounding structures. The coil serves as a radiographic marker during subsequent surgical resection, facilitating accurate localization and minimizing the risk of nodule misidentification or incomplete excision.

Several studies have reported on the efficacy and safety of CT-guided coil localization in lung nodule resections. In a retrospective study by Donahoe et al. [4], the authors evaluated the outcomes of 63 patients who underwent coil localization prior to video-assisted thoracoscopic surgery (VATS) resection. They reported a success rate of 100% in the diagnosis of nodules, with only minimal complications observed. Similarly, Wang et al. [5] conducted a prospective study involving 31 patients with a total of 42 subcentimeter ground-glass nodules and found that all localization procedures were successful.

The advantages of CT-guided coil localization include its ability to accurately target small or deep-seated nodules, its real-time visualization capabilities, and its potential to reduce surgical time and minimize tissue trauma [4,5]. However, despite these potential benefits, there remains a need for further investigation into the efficacy and safety of this technique across different clinical settings. Therefore, the present study aims to assess the efficacy and safety of CT-guided coil localization in lung nodule resections.

## Materials and methods

Study design and settings

This retrospective observational study was conducted at the National University Hospital in Singapore. The study evaluated the rates of successful localization, procedural complications, and postoperative length of stay for patients who underwent CT-guided coil localization and subsequent lung nodule resections. The study period spanned from 2015 to 2021.

Patient selection

A total of 55 patients who underwent CT-guided coil localization and subsequent lung nodule resections were included in the study. The patients were selected from the patient database at the National University Hospital, Singapore, during the specified study period. Inclusion criteria comprised patients with pulmonary nodules requiring surgical resection who underwent CT-guided coil localization for preoperative marking. Patients with contraindications for coil localization or incomplete data were excluded.

CT-guided coil localization

CT-guided coil localization was performed to mark the targeted pulmonary nodules prior to resection. The procedure was conducted under the guidance of CT imaging. The steps involved in the CT-guided coiling procedure included patient positioning and preparation, precise localization of the target nodule using CT imaging, percutaneous insertion of coils under CT guidance to mark the nodule, and confirmation of adequate coil placement and visualization of the target area. Following CT-guided coil localization, VATS was employed for the resection of the marked nodules.

Data collection

The data were collected retrospectively from electronic medical records and imaging reports. The collected variables encompassed the medical record number, lesion site, use of additional markers, number of coils, sex, background cancer, CT features, dimensions of the nodule, distance to the pleura, successful localization or not, postoperative histology, and complications.

Statistical analysis

Statistical analysis involved the use of descriptive statistics to summarize patient and nodule characteristics, coiling procedure details, the VATS resection procedure, and outcomes. Continuous variables are presented as means with standard deviations, while categorical variables are expressed as frequencies and percentages. All analyses were performed using Statistical Package for Social Sciences version 26 (IBM Corp., Armonk, NY).

Ethical considerations

This study was conducted in accordance with the ethical principles outlined in the guidelines of the National University Hospital, Singapore, and adhered to the principles of the Declaration of Helsinki. The study protocol was approved by the Institutional Review Board (IRB) of the National University Hospital (Approval Number: 2021-251). As this was a retrospective study utilizing existing patient data, the need for informed consent was waived by the IRB.

## Results

Patient characteristics

A total of 55 patients (27 males and 28 females) were included in the study. The smoking status of the patients was as follows: 6 (10.9%) were smokers, 38 (69.1%) were nonsmokers, and 11 (20.0%) were ex-smokers. Among the patients, 42 (76.4%) had a history of previous malignancy, with 9 of them having a history of lung malignancy and the remaining patients having nonlung malignancies (Table 1).

**Table 1:** Patient characteristics

Characteristic	Frequency	Percentage
Smoking status		
Smokers	6	10.9%
Nonsmokers	38	69.1%
Ex-smokers	11	20.0%
History of malignancy		
Lung malignancy	9	16.4%
Non-lung malignancy	33	60.0%

Nodule characteristics

A total of 58 pulmonary nodules were coiled. The distribution of coiled nodules according to their location was as follows: 15 (25.9%) in the right lower lobe, 10 (17.2%) in the right upper lobe, 3 (5.2%) in the right middle lobe, 15 (25.9%) in the left lower lobe, and 15 (25.9%) in the left upper lobe. The dimensions of the nodules were measured as 8.4 ± 3.2 mm by 10.1 ± 4.0 mm. The average distance from the pleura was 10.9 ± 9.0 mm. The morphology of nodules revealed that 25 (43.1%) were ground-glass opacities, 2 (3.4%) were cavitary nodules, and 31 (53.4%) were solid nodules (Table 2).

**Table 2:** Nodule characteristics

Characteristic	Frequency	Percentage
Nodule location		
Right lower lobe	15	25.9%
Right upper lobe	10	17.2%
Right middle lobe	3	5.2%
Left lower lobe	15	25.9%
Left upper lobe	15	25.9%
Nodule morphology		
Ground-glass opacity	25	43.1%
Cavitary nodules	2	3.4%
Solid nodules	31	53.4%

Surgical procedure

During the surgical procedure, the coiled nodules were resected. The resected volume averaged 91.1 ± 76.0 cm^3^, and the resection margin measured 9.5 ± 7.3 mm. Frozen section analysis was performed in 11 cases. Failed localization was observed in only two cases (3.6%) out of the 55 patients. Histological analysis of the coiled nodules revealed that out of the 55 cases, 14 (25.5%) were diagnosed as benign pathology, 21 (38.2%) as lung cancer, and 20 (36.4%) as metastatic lesions.

Complications and length of hospital stay

Among the patients, 12 (21.8%) experienced complications related to the procedure. These complications included 1 (1.8%) hemothorax, 4 (7.3%) persistent air leaks, 2 (3.6%) pneumothoraxes, 1 (1.8%) pleural effusion, 3 (5.5%) cases of pneumonia, and 1 (1.8%) atelectasis. All complications were managed appropriately. It is important to note that these complications were primarily related to the VATS procedure rather than the coil placement. The average length of postoperative hospital stay was 6.7 ± 15.9 days.

## Discussion

In this retrospective study, we evaluated the efficacy and safety of CT-guided coil localization in lung nodule resections. Our findings demonstrate promising outcomes, supporting the use of this technique as an effective and safe approach for the preoperative marking of pulmonary nodules.

Alternative techniques for preoperative localization of small peripheral pulmonary nodules have been described, including the use of a hookwire [6,7]. While the hookwire technique has shown high success rates, it is associated with complications such as dislodgement [8]. In our study, we employed CT-guided coil localization, which demonstrated a high success rate, with failed localization observed in only 3.6% of cases. The use of CT-guided coil localization offers advantages such as precise marking, minimizing the risk of incomplete resections, and potentially reducing complications associated with hookwires.

The success rate of CT-guided coil localization in our study was high, with successful localization achieved in 96.8% of cases. This high success rate is consistent with previous studies that have reported successful localization rates ranging from 90% to 100% [9-13]. The accurate marking of the targeted nodules enabled precise identification during subsequent surgical procedures, facilitating successful resection and minimizing the risk of incomplete resections.

Despite the occurrence of procedural complications, the manageable nature of these complications and the absence of major adverse events highlight the overall safety of the CT-guided coil localization technique. While our study identified complications such as persistent air leaks, pneumothoraces, and pneumonia, it is important to note that these complications were primarily related to the VATS procedure rather than the coil placement itself. The observed complications were successfully managed through vigilant monitoring and timely intervention, underscoring the effectiveness of our approach in ensuring patient safety. These findings are consistent with previous studies that have reported similar rates of complications associated with VATS procedures [4,14,15].

Comparing our results to the literature [5,9,12,14], our study reaffirms the efficacy and safety of CT-guided coil localization in lung nodule resections. The success rate of localization, accuracy in identifying malignant nodules, and manageable complication rates align with previous reports. This consistency supports the robustness and reliability of this technique across different patient populations and healthcare settings.

Despite the promising findings, several limitations should be acknowledged. First, the retrospective nature of our study introduces inherent biases and limitations associated with data collection and selection. Second, the sample size was relatively small, which may limit the generalizability of our results. Additionally, the study was conducted at a single center, potentially limiting the external validity of our findings. Future studies with larger sample sizes and multicenter designs are warranted to further validate our results and explore potential variations across different settings.

## Conclusions

In conclusion, our study demonstrates that CT-guided coil localization is an effective and safe technique for the preoperative marking of pulmonary nodules. With a high success rate of localization, accurate identification of malignant nodules, and a manageable complication profile, CT-guided coil localization offers a valuable approach to guide precise resection and facilitate optimal treatment planning. Despite certain limitations inherent to our retrospective study design and single-center setting, our results contribute to the growing body of evidence highlighting the clinical utility of CT-guided coil localization in lung nodule resections.
